# Unexpected complication of oesophagoscopy: iatrogenic aortic injury in a child

**DOI:** 10.5830/CVJA-2016-015

**Published:** 2016

**Authors:** Orhan Tezcan, Sinan Demirtas, Celal Yavuz, Menduh Oruc, Oguz Karahan, Mahir Kuyumcu

**Affiliations:** Department of Cardiovascular Surgery, Medical School of Dicle University, Diyarbakir, Turkey; Department of Cardiovascular Surgery, Medical School of Dicle University, Diyarbakir, Turkey; Department of Cardiovascular Surgery, Medical School of Dicle University, Diyarbakir, Turkey; Department of Chest Surgery, Medical School of Dicle University, Diyarbakir, Turkey; Department of Chest Surgery, Medical School of Dicle University, Diyarbakir, Turkey; Department of Anesthesiology, Medical School of Dicle University, Diyarbakir, Turkey

**Keywords:** oesophagoscopy, complication, aortic injury

## Abstract

**Introduction:**

Oesophagoscopy is usually a safe procedure to localise and remove ingested foreign bodies, however, unexpected complications may develop during this procedure. In this case report we discuss iatrogenic aortic injury, which developed during oesophagoscopy, and its immediate treatment.

**Case report:**

A six-year-old male patient was admitted to hospital with symptoms of having ingested a foreign body. Oesophagoscopy was carried out and the foreign body was visualised at the second constriction of the oesophagus. During this procedure, profuse bleeding occurred. Subsequently, a balloon dilator was placed to control bleeding in the oesophagus. Thoracic contrast tomography revealed thoracic aortic injury. Open surgical aortic repair was immediately carried out on the patient and the oesophageal hole was primarily repaired. The patient was discharged on postoperative day 15 with a total cure.

**Conclusion:**

Although oesophagoscopy is a safe, easily applied method, it should be kept in mind that fatal complications may occur during the procedure. This procedure should be done in high-level medical centres, which have extra facilities for managing complications.

## Introduction

Oesophagoscopy is an effective diagnostic and treatment method for oesophageal pathologies, with 0.03 to 17% complication rates.^1^,^2^ Perforation and bleeding are the most important complications of this procedure. Previous reports have claimed that therapeutic interventions with oesophagoscopy present more risks with regard to complications, than other diagnostic procedures.^1^,^2^

Ingestion of a foreign body into the oesophagus has serious potential for perforation.^3^ Oesophagoscopy strategies can be used both for detecting the location of the foreign body and for removal of it. However, it should be borne in mind that treatment with oesophagoscopy has the potential for further aortic injury if sufficient pre-operative evaluation of the anatomical and pathological status is not done.^3^ In this study, we present a case of aortic injury during oesophagoscopy in a patient with foreign body ingestion.

## Case report

**Fig. 1:**
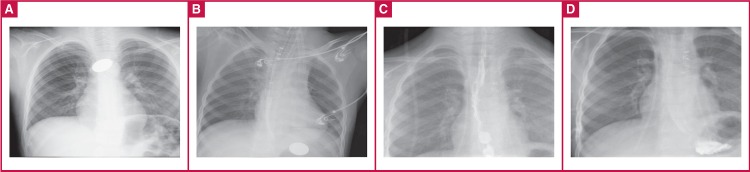
A. Chest radiography showing the first position of the ingested coin. B. Reposition of the coin in the stomach and visualisation of the achalasia balloon. C, D. Oesophageal radiographs on postoperative day 12.

A six-year-old male patient was admitted to hospital with dysphagia. Chest radiograms revealed the image of a coin at the second constriction of the oesophagus (Fig. 1A).

**Fig. 2:**
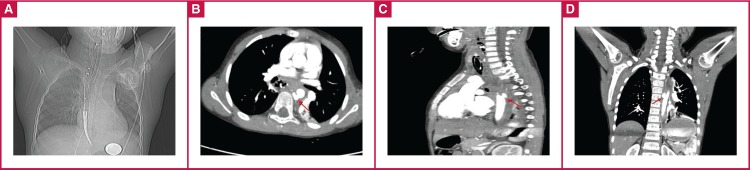
A. Achalasia balloon placed in the oesophagus. B. Contrast extravasation (red arrow) from aortic rupture (axial view). C. Contrast extravasation (red arrow) from aortic rupture (sagittal view). D. Contrast extravasation (red arrow) from aortic rupture (coronal view).

Rigid oesophagoscopy was carried out on the patient under general anaesthesia. Copious bleeding was noted during removal of the foreign body, so flexible oesophagoscopy was used. The injury site could not be determined due to severe haemorrhage. Because the blood pressure was dropping rapidly (60/30 mmHg), an achalasia balloon (polyethylene balloon) dilator (Fig. 1B), (Fig. 2A) was placed in the oesophagus to control the bleeding.

The haemoglobin level was 5 g/dl and three units of erythrocyte suspension replacement were administered immediately. After controlling the bleeding with the balloon dilator, contrast tomography (CT) was carried out. The coin was visualised in the stomach during the chest radiography (Fig. 1B),(Fig. 2A). Contrast extravasation revealed it near the descending aorta (crossing site of oesophagus) (Fig. 2A,B,C).

**Fig. 3:**
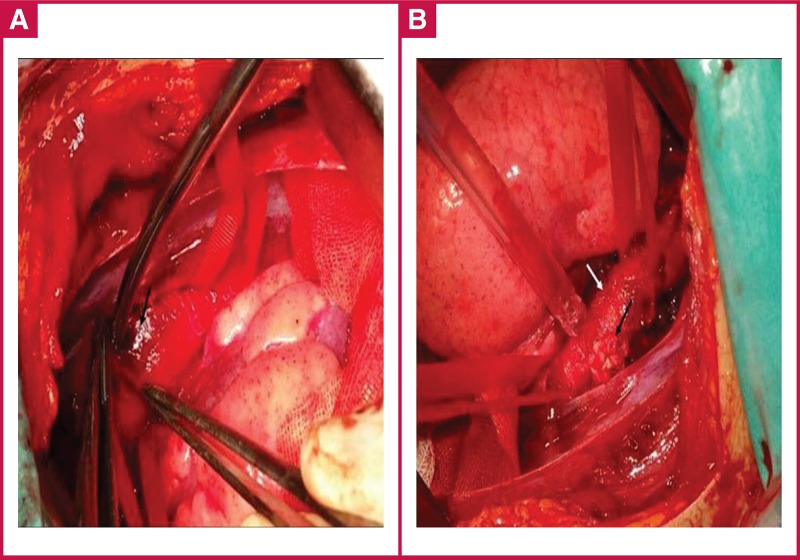
A. Adventitial haematoma from the aortic injury. B. Aortic injury repaired with pledgeted suture.

We consulted with a cardiovascular surgeon and immediate surgery on the descending aorta was planned. The patient was taken to the theatre for aortic repair. A left posterolateral thoracotomy was carried out on the patient for surgical exploration of the descending aorta. The injured site of the aorta was detected (Fig. 3a) and then repaired primarily with pledgeted sutures (Fig. 3B).

Just below the damaged aorta, a 1-cm oesophageal injury was detected. After dissection of the parietal pleura, the oesophagus was primarily repaired to avoid development of an aortooesophageal fistula. The patient was taken to the intensive care unit after the operation.

A graph of oesophageal passage was done on postoperative day 12 with iohexol (Omnipaque®, Nycomed, Oslo, Norway) contrast agent and no leakage was observed (Fig. 1C,D). The patient was discharged uneventfully on the 15th day after the operation.

## Discussion

We could not find any cases in the literature reporting aortic rupture during rigid oesophagoscopy. Therefore we report on this case with a view to preventing such complications in other patients.

Foreign body ingestion is frequently seen in early childhood. Peristaltic transmission of these foreign bodies to the stomach may be challenging due to anatomical constriction of the oesophagus.^4^,^5^ In these cases, endoscopic (10–20%) or surgical (1%) removal may be required.^4^,^5^ However, both the foreign body and the endoscope used for removal of the object could lead to aortic wall injury.^3^,^5^

Incidence of oesophageal rupture is reported at 0.1 to 1.9% during rigid oesophagoscopy.^6^ Aorto-oesophageal fistula is a rare but fatal (40–60%) complication of foreign body ingestion. The common site of aortic injury is at the second constriction of the oesophagus, which has a relatively narrow lumen due to the cross-over of the left primary bronchus and aortic arch.^3^

Aortic rupture of the oesophagus usually has a fatal course, particularly with spontaneous rupture of the aorta. He *et al* reported sudden death due to ruptured pseudo-aneurysms into the oesophagus.^7^ However, iatrogenic injuries of the aorta can be more easily controlled than unforeseen events. Therefore, pre-operative staging of the condition and planning of the procedure is important to avoid fatal outcomes.

Similar injuries have been reported with other thoracic interventions, such as spinal instrumentation and surgical and endovascular aortic repair techniques described for treatment of complications.^8^ However, direct aortic injury during oesophagoscopy is not reported as frequently as other complications. The majority of reports mention oesophageal rupture due to foreign body removal with oesophagoscopy.^1^,^2^

Oesophageal perforations may cause fatal outcomes due to mediastinitis and fulminant sepsis, which could prevent oesophageal repair.^1^,^2^ However, in combination with aortic rupture, this may become an emergency situation. An aortooesophageal fistula will usually occur after neglect of an ingested foreign body, as the oesophagus compresses the object, which irritates the oesophageal wall. This condition may develop over time, but iatrogenic aorto-oesophagial rupture occurs suddenly and progresses quickly to haemorrhagic shock.^7^,^9^ Therefore 80 to 90% of aortic injuries are fatal, and immediate aortic repair is important for survival.^7^ In our case, immediate aortic surgery was carried out after controlling the bleeding with a polyethylene balloon and CT detection of the injury.

Contrast CT scan is a suggested imaging technique for detecting the site of aortic injury and its relationship with surrounding structures.^3^ However, angiography can be undertaken for determining aortic pathology and treatment of the injury with endovascular techniques.^3^,^8^ Repair with an endovascular graft is a safer option for acute aortic injury. However, there is limited experience with this procedure in the paediatric population and natural progression of the stent is not fully known. Therefore, open surgical repair is the preferred technique for paediatric patients with aortic injury.^8^,^10^

In accordance with the therapeutic opportunities of the surgical centre, an appropriate method should be chosen and an immediate treatment protocol should be determined. In our case, we decided on open repair with simultaneous intervention on the aortic injury and oesophageal rupture.

## Conclusion

Aortic wall injury may occur during oesophagoscopy. Balloon dilatators may be helpful to control bleeding and secure time for surgical repair. Open aortic repair may ensure patient survival and it allows simultaneous oesophageal repair.
